# Expression of GDAP1 Gene Correlates With Alcohol Deprivation Effect

**DOI:** 10.1111/gbb.70022

**Published:** 2025-04-18

**Authors:** Rona Yarimay, Dominik K. E. Beyer, Annakarina Mundorf, Nadja Freund

**Affiliations:** ^1^ Division of Experimental and Molecular Psychiatry, Department of Psychiatry, Psychotherapy and Preventive Medicine LWL University Hospital, Ruhr‐University Bochum Bochum Germany; ^2^ Institute for Systems Medicine and Department of Human Medicine MSH Medical School Hamburg Hamburg Germany

**Keywords:** addiction, hippocampus, real‐time PCR, rodent models, voluntary drinking

## Abstract

Alcohol addiction is a widespread disease, and the exact causes and consequences are still not fully determined. Neurotransmitters and neuronal circuits are not only the target structure of alcohol and responsible for its direct effects but also play a central role in the development of addiction. A gene that has been linked to alcohol use disorder in recent studies is the *ganglioside‐induced differentiation‐associated protein 1 (GDAP1)* gene. The present study focuses on the hippocampus, a brain region particularly vulnerable to alcohol and rich in *Gdap1* gene expression. Using an established drinking model, alcohol drinking behavior was induced in adult male Long Evans rats. After 6 weeks of voluntary alcohol intake, followed by 2 weeks of deprivation, the animals were divided into two groups based on the alcohol deprivation effect (ADE). *Gdap1* gene expression was measured with real‐time PCR in the hippocampus. Results reveal significantly decreased mRNA expression in the high ADE group compared to the low ADE group. This decrease was specifically detected within the cornu ammonis 3 (CA3) region. *Gdap1* expression in this region also negatively correlated with ADE in all animals. Taken together, results indicate that *Gdap1* might not only be associated with alcohol consumption but might even play a role in alcohol dependence.

## Graphical abstract

After 6 weeks of alcohol acquisition, male mice underwent alcohol deprivation for 2 weeks. Afterwards, the alcohol deprivation effect (ADE) was measured, and animals were divided into two groups accordingly. The group with low ADU significantly differed in GDAP1 mRNA expression in the hippocampus compared to the high ADU group.

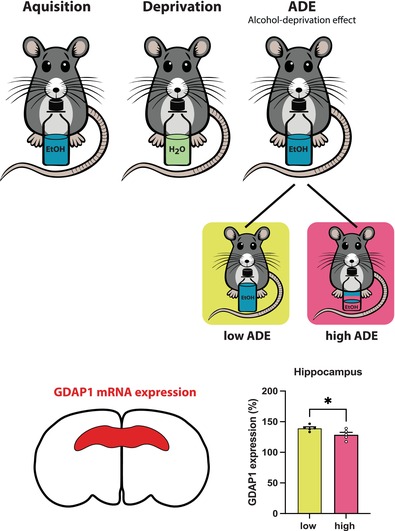

## 1 Introduction

The harmful use of alcohol is linked to over 200 health conditions, including liver diseases, road injuries and violence, cancer, cardiovascular diseases, and suicides [1]. The main effect of alcohol, however, is on the central nervous system. These effects are diverse and not yet fully understood, but a damaging impact has been proven by several studies. Long‐term and excessive alcohol consumption also causes changes in brain structures [2]. Structures that are particularly affected by neuronal changes and are vulnerable to alcohol exposure are the cerebral cortex, especially the prefrontal cortex, the limbic system, including the hippocampus, and the cerebellum [3]. The influence of alcohol on the hippocampus has often been the subject of research, but much remains unclear.

The hippocampus appears to be one of the brain structures that is affected through various mechanisms by alcohol consumption. Several studies found a significant reduction in volume in humans with alcohol use disorder (AUD) [4, 5, 6]. The volume reduction can partly be attributed to an inhibition of hippocampal neurogenesis caused by alcohol, which has been demonstrated in both humans and rats [7]. Additionally, animal studies have shown decreased hippocampal cell proliferation and increased cell death after chronic alcohol exposure [8, 9].

Genetic factors contribute to 40%–60% of the risk for harmful alcohol use [10]. Besides genetic factors, environmental influences also make a major contribution, including the availability of alcohol, attitudes toward drinking, levels of stress, and regulatory frameworks [10]. Genetics and environmental influences are interconnected through epigenetics. In 2014, an epigenome‐wide association study (EWAS) was conducted to study alcohol addiction. Philibert and colleagues [11] examined DNA methylation in peripheral mononuclear cells from subjects before and after short‐term alcohol treatment and identified the 30 most significantly associated genes, including the ganglioside‐induced differentiation‐associated protein‐1 (*GDAP1*) gene, linking this gene to alcohol dependency for the first time. During the past years, the *GDAP1* gene gained more attention from neurological research due to its significant role in the Charcot‐Marie‐Tooth (CMT) disease, an inherited peripheral neuropathy [12]. CMT is a neuromuscular disorder characterized by the degeneration of peripheral nerves leading to muscle weakness and sensory loss in distal limbs, feet, and hands [13]. Studies using *Gdap1* knockout mice (*Gdap1* ^−^/^−^) have shown that a deficiency of *Gdap1* causes calcium depletion and changes in the mitochondrial network biology and mitochondria‐endoplasmic reticulum interaction [14]. Mitochondrial dysfunction, in turn, can trigger oxidative stress and subsequently lead to neuroinflammation [15], as is the case in both the axonal and demyelinating forms of CMT [16, 17]. *GDAP1* is ubiquitously expressed in the central nervous system in the grey matter of the brain and spinal cord and especially in large neurons like hippocampal pyramidal neurons as well as in smaller neurons like hippocampal interneurons [18]. It was also shown in the peripheral neuronal system expressed by Schwann cells [19]. On the subcellular level, *GDAP1* is located in the mitochondrial outer membrane (MOM) as a novel regulator of the mitochondrial network and in mitochondrial‐associated membranes (MAMs) [20]. Studies have shown that an overexpression in *GDAP1* affects overall mitochondrial activity, leads to alterations in the morphology, and induces mitochondrial fission and fragmentation [14, 19, 21].

Based on the results by Philibert et al. [11], another study investigated the role of *GDAP1* in an independent cohort using whole blood cells, proving a hypomethylation of *GDAP1* in patients with alcohol addiction. Using the AUDIT and the GSI score as an indicator for alcohol dependency, they found a negative association between these scores and *GDAP1* methylation as well as a negative association between *GDAP1* methylation and the years of alcohol dependency [22]. In addition, there was a decrease in *GDAP1* hypomethylation after a short‐term alcohol treatment program, which points toward a potential role of *GDAP1* hypomethylation as a biomarker for therapy outcome [22].

Downregulation of *GDAP1* was, in addition, revealed in a study by Edmiston et al. [23] who used cDNA microarray data to identify genes in peripheral blood leucocytes from adults with and without chronic obstructive pulmonary disease (COPD), a disorder mainly caused by tobacco smoking [23]. Animal studies confirm the involvement of GDAP1 and tobacco smoking. Mundorf et al. [24] compared a group of 11 female mice, exposed to the smoke of five cigarettes daily for 5 weeks, to a non‐exposed group. Results revealed region‐dependent effects on GDAP1 protein expression with an increased level of protein in the hippocampal DG and a decreased level in the CA1 region using immunohistochemical staining [24]. These findings are important since there is a high comorbidity between alcohol dependence and tobacco dependence. A study from the 1980s in the USA estimated that more than 80% of alcohol addicts are also heavy smokers, and on the other side, about 40% of heavy tobacco smokers meet the criteria for alcohol dependence [25]. Although the complete mechanism is unexplained, some theories try to clarify the correlation between these two substances, including nicotine's and alcohol's mutually rewarding system, nicotine counteracting some adverse effects of alcohol, and a common genetic susceptibility while sharing some of the same genetic determinants [26].

The aim of the present study was to investigate the correlation between *Gdap1* in the hippocampus and alcohol drinking without any additional exposure to nicotine. Beginning with a phase of voluntary alcohol intake, animals were deprived of alcohol, followed by a phase of increased alcohol consumption and preference, the alcohol deprivation effect (ADE). During this phase, rats in long‐term alcohol self‐administering experiments show compulsive, uncontrolled drug‐seeking and drug‐taking behavior. The animals show signs of tolerance development, physical and psychological signs of withdrawal, and stress‐induced drinking, which is consistent with the diagnostic criteria of alcoholism in humans according to the DSM‐IV [27, 28]. At the neuromolecular level, the NMDA receptor system seems to play a key role as it is inhibited by alcohol consumption, leading to excessive excitability during abstinence in the central nervous system, which can cause craving and lead to relapse [29, 30]. Based on the studies by Philibert et al. [11], Brückmann et al. [22], and Mundorf et al. [24] we hypothesized that animals with a strong ADE show high expression of *Gdap1* in the hippocampus due to hypomethylation of the corresponding gene.

## 2 Material and Methods

### 2.1 Animals

A total of *n* = 10 adult, male Long Evans rats were obtained from Charles River Laboratories (Sulzfeld, Germany). Long Evans Rats are commonly used for alcohol research since they show a higher preference for alcohol than other strains, and especially cycles of abstinence induce high levels of ethanol consumption [31]. Rats were kept in a standard cage with food and water ad libitum in constant temperature and humidity conditions (22°C ± 2°C and 55% ± 25%) on a 12‐h light/12‐h dark cycle (light period 23:00–11:00). The animals were allowed to acclimate to the animal facility for 1 week before starting the experiments. Body weights were measured weekly. Only males were used to avoid an influence of the estrous cycle of female rats on the hippocampus [32]. All experiments were carried out in agreement with the principles regarding the care and use of animals adopted by the German Animal Welfare Law for the prevention of cruelty to animals after approval by the LANUV (Landesamt für Natur, Umwelt und Verbraucherschutz Northrhine‐Westfalia; Az: 84‐02.04.2017.A267).

### 2.2 Voluntary Oral Alcohol Intake

The voluntary alcohol drinking behavior and the ADE were used as a model for relapse‐like drinking behavior [33, 34].

At the beginning of the experiment, while still in group housing, the rats were given three bottles of tap water to get used to the presence of three bottles and to avoid the preference to use only one bottle for drinking. After a week of handling, they were put in single housing and were given access to 5% and 20% ethanol solutions in addition to tap water ad libitum. The alcohol solutions were diluted from 99.8% ethanol with tap water.

**FIGURE 1:**
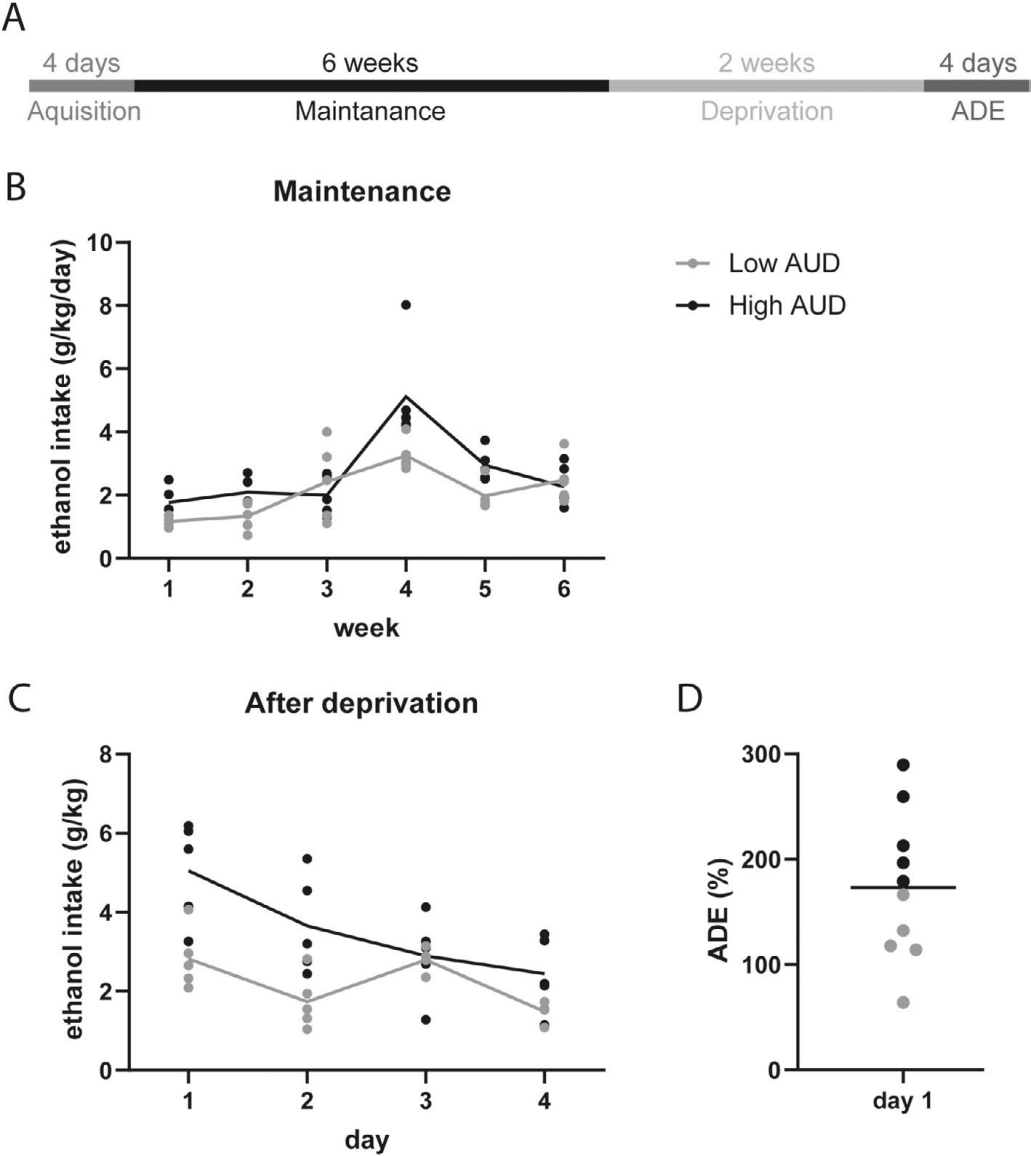
Voluntary alcohol consumption. After 6 weeks of voluntary alcohol consumption (A) animals consumed alcohol at relatively stable levels (B). After 2 weeks of deprivation of alcohol (A), a deprivation effect (ADE) could be observed (C). According to the ADE two groups were built (D). *p* < 0.05.

For the first 4 days, the alcohol consumption was measured daily to observe the acquisition. The following 6 weeks, the bottles were weighed once a week and the positions changed twice a week to avoid side preferences. The ethanol solutions were replaced weekly to maintain a constant alcohol concentration. After this phase of maintenance, alcohol was deprived for 2 weeks and afterward measured daily for 4 days to assess the ADE (Figure 1A).

### 2.3 RNA Isolation and Real‐Time PCR

For rtPCR quantification of *Gdap1*, rats were sacrificed on the fourth day after deprivation, and the hippocampus from each rat brain was dissected and separated into CA1, CA2, CA3, and DG of each hemisphere, according to the rat brain atlas [35]. To account for potential hemisphere‐specific differences [36, 37], hemispheres were analyzed separately. RNA isolation was performed using the NucleoSpin TriPep Kit (Macherey‐Nagel, Düren, Germany) with slight modifications, meaning that 60 μL DNA Elute solution was used for DNA elution and 40 μL of RNase‐free water was added to obtain RNA. After the quantification and quality assessment, RNA was reverse transcribed to cDNA using the High‐Capacity RNA‐to‐cDNA Kit (Thermofisher Scientific, Darmstadt, Germany) according to the manufacturer's protocol. Experiments for establishing *Gdap1* rtPCR revealed a minimum of 20 ng RNA needed per sample to detect stable results. Therefore, 20 ng cDNA was used to measure *Gdap1* mRNA levels for each region and hemisphere separately. For rtPCR analysis, TaqMan Gene Expression Master Mix (Thermofisher) and TaqMan gene expression assay for *GDAP1* (Rn01749152_m1) as well as for Glyceraldehyde‐3‐phosphate dehydrogenase (Rn01775763_g1) and Actin, beta (Rn00667869_m1) serving as housekeeping genes were used. The CFX Connect Real‐Time PCR System (Bio‐Rad) was used according to the manufacturer's protocol. All samples and gene assays were assayed in duplicates.

### 2.4 Analysis

Based on the weight changes of the respective bottles, the amount of consumed alcohol in grams (g) per day and weight in kg of the animal were calculated. For measuring ADE, the percentage of the amount consumed on the first day after deprivation and the average of the consumption on the last maintenance week before the deprivation were used.

RNA levels were quantified by the number of cycle thresholds (Delta CT method). The ∆Ct value of each sample was calculated by subtracting the mean Ct value of the housekeeping genes from the mean Ct value of *Gdap1*. To account for individual plate differences, a reference sample taken from the uterus was run on all plates, as the uterus is one of the few peripheral tissues showing high *Gdap1* expression. Values were calculated as percentages compared to the respective ∆Ct value of the uterus sample.

## 3 Results

During maintenance, animals consumed on average 2.4 (±0.2) g/kg/day alcohol with a minimum of 1.78 g/kg/day and a maximum of 3.74 g/kg/day (Figure 1B). After 2 weeks of deprivation, the average consumption was highest on the first day at 3.9 (±0.5) g/kg and lowest on the fourth day at 1.97 (±0.3) g/kg (Figure 1C). ADE was on average 173.2 (±22)% with a minimum of 64.2% and a maximum of 289.6%. The mean was set as the threshold, resulting in five animals with values above the mean (high ADE) and five animals below the mean (low ADE) (Figure 1D).

**FIGURE 2:**
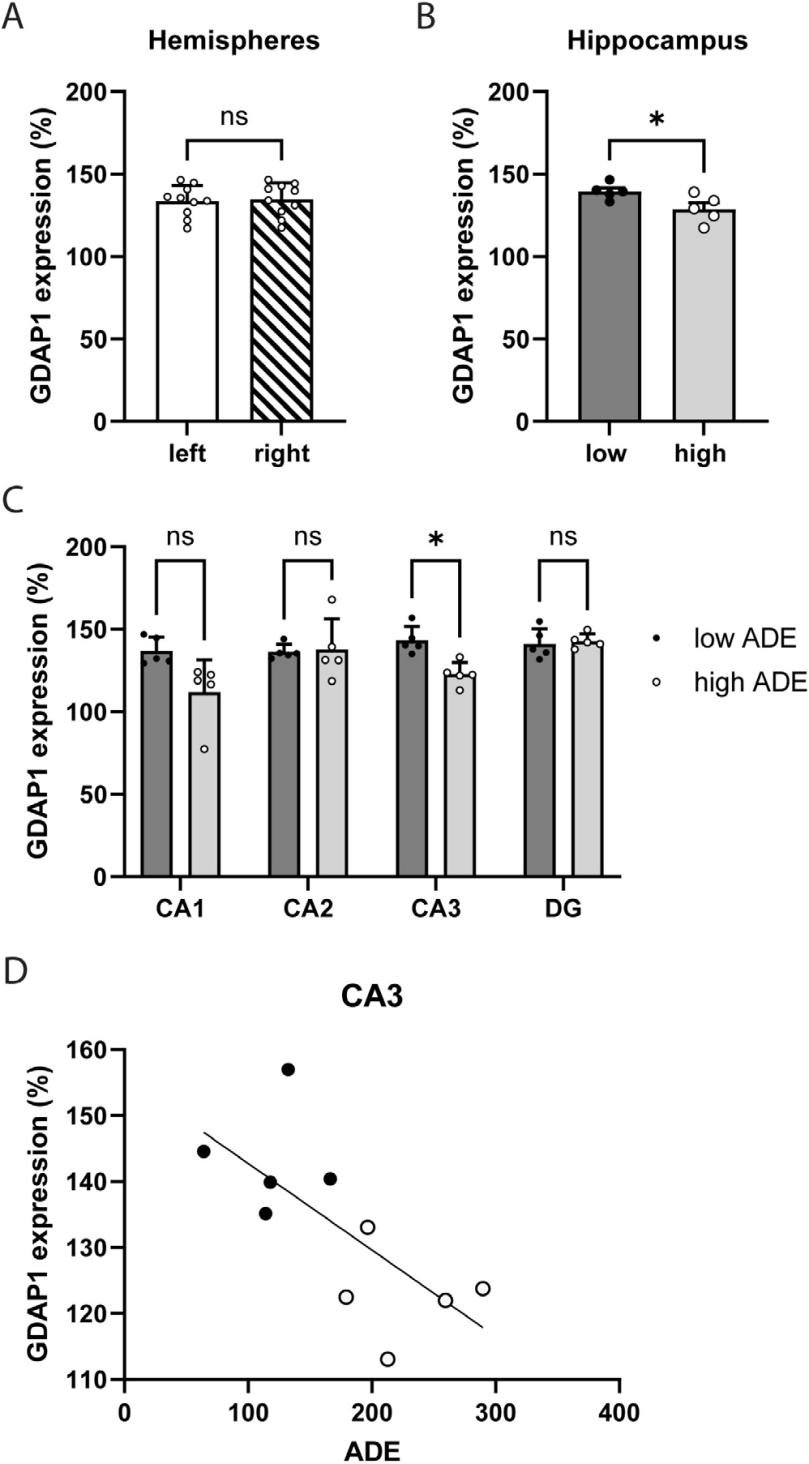
GDAP1 gene expression in hippocampus. No hemispheric expression differences could be detected (A). However, the high ADE group showed significantly lower expression compared to the low ADE groups (B). This effect was mainly driven by the CA3 region of the hippocampus (C). GDAP1 expression in CA3 even negatively correlated with ADE (D). *p < 0.05.

Gene expression for *Gdap1* in the left hippocampus was 133.6 (±3)% and 134.7 (±3.1)% in the right hippocampus (Figure 2A). As this difference was not significant *t*(9) = 0.37; *p* = 0.72; means between the hemispheres were calculated for each animal. Animals in the low ADE group showed an overall higher expression of *Gdap1* in the hippocampus compared to the high ADE group *t*(9) = 2.5; *p* = 0.038 (Figure 2B). When looking at different subregions of the hippocampus, the two groups significantly differed in the CA3 region *t*(8) = 4.2; *p* = 0.01 (Bonferroni corrected) but not in the CA1 *t*(8) = 2.6; *p* = 0.12, the CA2 *t*(8) = 0.16; *p* > 0.99 and the DG *t*(8) = 0.34; *p* > 0.99 (Figure 2C). Simple linear regression was then used to test if ADE predicted the expression of *Gdap1* in CA3. The overall regression reached significance (*R* ^2^ = 0.5, *F*(1,8) = 7.6, *p* = 0.025) indicating that ADE predicts the *Gdap1* mRNA expression in CA3 (Figure 2D).

## 4 Discussion

Using an established drinking model [33, 34], we were able to induce voluntary stable alcohol consumption in male adult Long Evans rats. After a withdrawal period of 2 weeks to induce alcohol craving, most animals did increase their consumption compared to the consumption during maintenance, indicating the occurrence of an ADE. However, the magnitude of ADE differed between individuals and allowed the separation into low and high ADE animals. Interestingly, high and low ADE animals differed in *Gdap1* RNA expression in the hippocampus, with higher expression in the low ADE group. This difference was particularly pronounced in the CA3 region of the hippocampus, with a negative correlation between ADE and *Gdap1* expression. Differences in the expression of *Gdap1* indicate an important role of this gene in alcohol dependency. In human cohorts [11, 22], *GDAP1* methylation differed between individuals with alcohol dependence and healthy subjects, and even correlated with the degree of dependence. When accounting for the amount of alcohol consumed 1 week prior to blood analysis, no influence on the *GDAP1* methylation pattern was found [22]. Nevertheless, overall amounts of alcohol consumed might correlate with the degree of dependency, and the assessment of consumed alcohol by questionnaires might be influenced by subjectivity. The present study allowed for precise individual control of the exact amounts of alcohol consumed. High and low ADE groups did not differ in the amounts of alcohol consumed during maintenance nor on the 2 days prior to brain tissue analysis. Therefore, the results confirm that *Gdap1* is not associated with overall consumption but with relapse‐like behavior, one sign of dependence.

In contrast to the hypothesis, *Gdap1* gene expression was negatively correlated with ADE. In the human cohorts, individuals with alcohol dependence showed DNA hypomethylation, which is assumed to lead to increased gene expression [11, 22]. One explanation could be the difference in tissue analyzed. While in this study, brain tissue, namely hippocampal regions, was investigated, others extracted DNA from peripheral mononuclear cells [11] or used whole blood samples [22]. Region‐specific effects on *Gdap1* expression after nicotine exposure have been reported previously [24]. Taken together, the role of *GDAP1* in dependence might rather be complex and associated with dysregulation than a simple up‐ or downregulation.

The fact that the hippocampus plays a prominent role is not surprising. The hippocampus is one of the brain structures particularly affected by alcohol addiction [3, 4, 37, 38, 39]. At the same time, the *GDAP1* protein is expressed in hippocampal pyramidal neurons and interneurons [18]. Alcohol‐induced effects specifically on the CA3 region have not yet been widely researched. Quantitative analysis of CA3 pyramidal cells and mossy fiber‐CA3 synapses (MF‐CA3) in rats showed a loss of pyramidal cells in animals both consuming and withdrawing alcohol, as well as a decline in MF‐CA3 synapses after 18 months of alcohol consumption [40]. A loss of pyramidal cells containing *Gdap1* could explain why the high ADE group contains less *Gdap1* than the low ADE group. Another study demonstrated a decrease in the number of pyramidal cells in both the CA3 region as well as the CA1 region [41], in which a slight but not significant decrease could be detected. Consistent with our results is a study that examined the transcriptional neuronal activity in the regions of the hippocampus in male rats. The neuronal activity was measured by the number of argyrophilic nucleolar organizer regions (AgNORs) in the DG, CA3, and CA1 during alcohol administration and after withdrawal and showed that in both groups the CA3 region was the most affected by alcohol [42].

Another explanation of the common mechanism between *GDAP1* and alcohol dependence could be neuroinflammation. A deficiency of *Gdap1* is known to lead to oxidative stress and thus to neuroinflammation and neurodegeneration via changes in mitochondrial function [14]. Chronic alcohol consumption also contributes to neuroinflammation through various mechanisms, including the production of reactive oxygen species by mitochondria, and thus leads to oxidative stress and neuronal cell damage [43]. Whether and to what extent these two triggering factors for neuroinflammation could synergize with one another needs further research.

For the interpretation of the present study, certain limitations have to be considered. Animals consumed the alcohol voluntarily and were divided into low‐ and high‐relapse‐like groups based on their ADE. Future studies should consider investigating *Gdap1* specifically in rodent models for addiction, for example, in selectively bred alcohol‐preferring or high‐alcohol‐drinking rats [44]. Separating groups according to more elaborate behavioral measurements like operant self‐administration paradigms with extinction and reinstatement [45] could, in addition, give more insights into the specific role of *Gdap1* in regulating dependence. It also has to be considered that the present study found a correlation between *Gdap1* expression and ADE but has no proof of causation. Future studies should investigate if a controlled manipulation of hippocampal *Gdap1* expression, for example, by using viral constructs, leads to altered alcohol consumption and behavior associated with dependence. It is important to note that this study exclusively used male animals. Research indicates that alcohol affects the hippocampus differently based on sex in both humans [46] and rodent models [47]. Consequently, female rats may exhibit different correlations between ADE and *Gdap1* expression. Additionally, the relatively small sample size should be taken into account when interpreting the results.

Studies with human patients collectively indicate that the *Gdap1* gene plays a role in alcohol dependence. In the present study, the expression of the *Gdap1* gene in the CA3 region of male adult rats correlated with the ADE, which reflects relapse‐like behavior. Interestingly, the amount of alcohol consumed did not correlate with *Gdap1* expression. These findings suggest that *Gdap1* is more closely associated with alcohol dependence rather than alcohol consumption. Further research is needed to elucidate the exact mechanisms, but *Gdap1* could potentially serve as a biomarker for addiction or the risk of addiction. Moreover, a deeper understanding of the mechanisms linking *Gdap1* to dependence could pave the way for new treatment options.

## Conflicts of Interest

The authors declare no conflicts of interest.

## Data Availability

The data that support the findings of this study are available from the corresponding author upon reasonable request.
